# Supplementary material for: Breaking barriers to completing genetic testing for inherited breast cancer among at-risk Black women using a community-based participatory research approach
Source: HGG Adv. 2026 Mar 17;7(2):100591. doi: 10.1016/j.xhgg.2026.100591 (PMC13071439; doi:10.1016/j.xhgg.2026.100591)
Supplement: Document S1. Materials S1 and S2 [file mmc1.pdf]

HGGA, Volume *7*

Supplemental information

Breaking barriers to completing genetic testing for inherited breast cancer among at-risk Black women using a community-based participatory research approach

Sarah H. Choi, Sanjana Ramesh, Shanequa Reed, Georgina Menyah, Pamela Ganschow, Vida Henderson, and Henry M. Dunnenberger

Supplemental Information Table of Contents

1. **Material S1 Semi-Structured Interview Guide**
2. **Material S2 Community Report of Study Findings**

Material S1 Semi-Structured Interview Guide

Introduction and Informed Consent:

Hello, my name is ________. I am a researcher with the Personalized Medicine team at Endeavor Health. I am calling about your scheduled interview for our research study, "Barriers and Facilitators to Genetic Testing in African American Women at Increased Risk for Inherited Breast Cancer." Is this time still good for you?

Thank you for agreeing to chat with me today. I appreciate your time and experience on this matter. The interview can last up to 60 minutes and I will be recording our session so I may go back and listen to it. Do you have any questions for me before we begin? (Wait for questions and answer as appropriate)

Before we begin, I would like to review the consent documents we provided you when we scheduled your call. [**review verbal informed consent document**].

[IF patient consents to continue on]

I'm going to start recording the audio so that we have a transcript of our conversation.

[Turn on recorder]

This is [interviewer name], and I am speaking with [participant's name]. When we create the transcription, we will replace your name with the code [participant ID#].

Do I have your permission to record this interview?

[Record response and begin the interview]

Overview of Interview:

To begin, I will explain a little bit about what we hope to accomplish today. During your last mammogram appointment, you completed an online questionnaire called the Breast Health Assessment (BHA), asking about your personal and family history of cancer. Based on your answers, you were recommended to get genetic testing to determine if you are at higher risk for inherited breast cancer. The goal of this interview is for us to learn more about your thoughts and feelings on the BHA and completing genetic testing at the time of your mammogram screening. We will start with very general questions and work on more specific questions. The most important thing to remember is that there is no single correct answer to our questions. We are genuinely interested in your thoughts and opinions about the BHA and completing genetic testing for inherited breast cancer. You are not required to answer any question you are uncomfortable with and your answers will not affect your healthcare in any way. Please feel free to share information as it comes to mind. Before we get started, do you have any questions for me?

Construct	Question:	Probes:	Notes:
**Knowledge**	What do you remember from the BHA family health history questionnaire?	Is there anything else?	
	Why did you decide to complete the BHA?<br><br>Did you find the questions easy or difficult to answer? Did you feel comfortable answering the BHA questions? Why?	Can you think of anything else? Can you share more about who in your family was diagnosed with cancer?	
	How did you feel after learning that you may have a higher-than-average risk of developing certain types of cancer, such as breast cancer?	Does anything else come to mind?	
	Have you ever been recommended to complete genetic testing before the BHA?	Has anyone ever mentioned genetic testing in your circle (family, friends, siblings, or anyone in your social circle)?	
	What comes to mind when you think about genetic testing services?	Don't worry, there is no right or wrong answer. Is there anything else that comes to mind?	
	How do you think genetic testing differs from other clinical services, such as mammograms?		
	Before today, what have you heard about genetic testing for inherited cancers such as breast cancer?	Is there anything else you have heard?	

**Describe genetic testing for IBC for the participant:** Thank you for sharing that information. For the purpose of this study, I am going to give you a little bit of background information about genetic testing for inherited breast cancer and why genetic testing is recommended for women with a personal and/or family history of cancer. If you have a family history of breast or other cancers, especially if there are young diagnoses (e.g., diagnosed before age 50) and multiple cases or a unique pattern of cancers on one side of your family, it is recommended that you get genetic testing to assess your risk for inherited cancer. This testing looks for changes or mutations in your genes that might increase your likelihood of developing certain cancers, including breast cancer. People identified as having a higher than average risk for developing inherited cancer on the BHA are offered genetic testing for genes related to common inherited cancers. If they decide to get genetic testing, they are given a saliva sample collection kit during their mammogram appointment to complete genetic testing.			
**Attitudes**	What are your initial thoughts about IBC?<br><br>What types of emotions do you have about IBC?<br><br>What comes up when you think about genetic testing for IBC?	What would be positive about genetic testing for breast cancer risk?<br><br>What concerns do you have about genetic testing for breast cancer risk?	Follow-up on positive and negative thoughts.
	How did you feel about the idea of completing genetic testing (i.e., providing a saliva sample) at the time of your screening mammogram?	Do you have any other thoughts or feelings?	
	How do you think completing genetic testing for inherited breast cancer might benefit you?	What other benefits can you think of?	Is there anyone else?
	What about your family members? Your community?		

	What do you think are the disadvantages or negative effects of genetic testing for inherited breast cancer?<br><br>Can you think of any other concerns?	Additional prompts –<br>- Timing of completing genetic testing<br>- Sample provision<br>- Privacy/Confidentiality<br>- Fear of receiving results	
	What would be important for you to know before you agree to provide a saliva sample for genetic testing?	Additional prompts –<br>- The testing laboratory<br>- The cost (insurance copay)<br>- The risk (EHR documentation, info shared with PCP)	

**Perceived Norms**	How do you think other people of African Descent feel about genetic testing?	How much do their opinions matter to you?	
		On a scale of 1 – 10, with 1 being, their opinions don't matter at all to a 10 being their opinions matter a lot, what would you say?	Why did you pick a [INSERT NUMBER]?
	How do you think your close friends and family would feel about you completing genetic testing for inherited breast cancer?<br><br>Would you inform family, friends, or children of testing?	How much do their opinions matter to you?	
		On a scale of 1 – 10, with 1 being, their opinions don't matter at all to a 10 being their opinions matter a lot, what would you say?	Why did you pick a [INSERT NUMBER]?
	Who do you believe would support your decision to complete genetic testing for inherited breast cancer risk?	What about your family and friends?<br>What is your relationship with this	Why do you believe that is?<br><br>How much do their

		person/people?<br>What about in your community?	opinions matter to you?
	Do you believe anyone would be against your decision to complete genetic testing for inherited breast cancer?	What about your family and friends?<br>What is your relationship with this person/people?<br>What about in your community?	Why do you believe that is?<br><br>How much do their opinions matter to you?
	Whose opinion might influence your decision to complete genetic testing?	What about your healthcare providers? Which one of your healthcare providers? Any organizations?	Why do these people's opinions matter to you?
	Do you know anyone who has completed genetic testing for cancer or other conditions?	Is it important that you know others who have completed genetic testing? Why or why not?	

**Self-Efficacy**	Why did you decide not to complete genetic testing at the time of your screening mammogram after filling out the BHA?<br><br>What made it difficult for you?<br><br>What concerns and/or emotions did you have when you made the decision not to complete testing during the mammogram visit?	Additional prompts – ask participants about:<br>- More time<br>- More information<br>- Healthcare provider input<br>- Presence of support person<br>- Sample collection process	

	What could have helped you to complete genetic testing at the time of your screening mammogram?	Additional prompts – ask participants about:<br>- Timing the sample provision<br>- Information<br>- Healthcare provider input – if so, how?	How?

**Salience of the behavior**	After our discussion today, how important is it to you to complete genetic testing for inherited breast cancer?	On a scale of 1 – 10, with 1 being not important at all to a 10 being very important, what would you say?	Would you like more information on how to complete genetic testing?
	How important do you believe it is for other African American women to complete genetic testing for inherited breast cancer? What about those who have been identified as high-risk?	On a scale of 1 – 10, with 1 being not important at all to a 10 being very important, what would you say?	Can you explain why you believe this?
Thank you so much for sharing your input throughout this interview. Your responses will help us figure out ways to break the barriers that women of African descent experience in accessing genetic testing services. It is important to us that we don't contribute to health inequities in this space. Before we go over your demographic information, we have one last question for you.			
**Wrap-up questions**	What advice do you have that might help us support other women of African descent women identified as high risk to complete genetic testing for inherited breast cancer?	Do you have any last comments or thoughts before we conclude the interview?	

Great, we really appreciate your participation in this study. Now, we will collect your demographic information. Your responses to these questions will be aggregated with the responses of other participants so that we can classify our sample.

1. What is your current age?

   a. 18-24
   b. 25-34

c. 35-44
d. 45-54
e. 55-64
f. Over 65

2. What gender do you identify with?
   a. Male
   b. Female
   c. Both male and female
   d. Neither male nor female
   e. Transgender male
   f. Transgender female

3. How do you describe your ethnicity?
   a. Hispanic/Latino
   b. Not Hispanic/Latino

4. How would you describe your racial background? (check all that apply)
   a. African American/Black
   b. Asian
   c. American Indian/Alaska Native
   d. Native Hawaiian/Pacific Islander
   e. White/Caucasian
   f. Other

5. What is your employment status?
   a. Full time
   b. Part time
   c. Student
   d. Retired
   e. Homemaker
   f. Looking for employment
   g. Not employed and not looking for employment
   h. Disabled

6. What is your current household income?

   a. Less than $25,000
   b. $25,000 to $49,999
   c. $50,000 to $74,999

d. $75,000 to $99,999
e. $100,000 to $149,999
f. $150,000 to $199,999
g. $200,000 to $249,999
h. $250,000 to $299,999
i. $300,000 or above

7. What is your current relationship status?

a. Married/Domestic Partner/Civil Union
b. Separated or Divorced
c. Widowed
d. Living with Partner
e. Single

8. Do you have biological children?
a. Yes (if yes, how many? ____________)
b. No

9. What is the highest grade or level of formal education you have completed?
a. Less than high school
b. High school
c. Some college (or certificate)
d. College graduate
e. Post graduate degree (MA, MS, PhD, MD, DO, etc.)

10. Do you currently practice a religion?
a. No
b. Yes
i. If yes, are you:
Protestant
Roman Catholic
Mormon
Orthodox Greek
Orthodox Russian
Jewish
Muslim
Buddhist
Hindu
Other

Thank you so much for your time. Before we conclude our discussion, is there anything else that you think is important for me to know?

Great, thank you again for your participation. In terms of next steps, you will receive a $50 Visa gift card as an appreciation for being in this research study. The gift card will be sent to you in the mail with a USPS Receipt of Delivery form in which you will acknowledge the receipt of the gift card by signing and returning the form. We do need to collect a little more information from you to process payment.

What is your phone number?
What is your email address?
What is your physical mailing address?

Once the study findings are analyzed, do we have your permission to contact you and send you a community report of the de-identified findings and conclusions from the study?

Wonderful, thank you so much for your time, we really appreciate it.

Material S2 Community Report of Study Findings

Breaking Barriers to Genetic Testing: What We Learned Together

Community Report of Research Findings

The **Breast Health Assessment (BHA)** is an electronic family history tool administered before routine mammograms to identify women at higher risk for inherited breast cancer and increase access to guideline-recommended genetic testing (GT).

***Why* We Did This Study:** Preliminary data revealed that women of African descent who screen high risk and consent to GT via the BHA have a low GT completion rate.

***What* We Did:** Partnered with community members using a community-based participatory research approach to explore barriers & facilitators to GT among at-risk women of African descent

- Designed a qualitative interview study
- Interviewed 12 participants
- Reviewed and interpreted interview findings

***What* We Heard:** Most participants wanted to complete GT and understood its importance for their health and their families. But real-world barriers got in the way.

Process Issues: 50% of participants never received a saliva collection kit for GT

Poor Follow-Up: Some who received kits were not confident to complete next steps

Lack of "Warm Hand-off": Participants wanted an interpersonal touchpoint for support

Cultural Barriers Not An Issue: Mistrust and limited knowledge had no impact on decision

***What* Can Be Done:** Through community input, we identified actionable strategies to address existing gaps in the BHA workflow and improve equitable access to GT

Clear Communication: Reach out before and after appointments to set expectations, and use culturally relevant messaging when providing supportive information

Interpersonal Support: Create more opportunities for human connection

Community Involvement: Continue to work together—because your voices are the key to designing and implementing programs that work

Thank YOU! We deeply appreciate your time, honesty, and trust in sharing your experiences with us.

Sarah H. Choi, MGCS, CGC
(Principal Investigator)
Endeavor Health

Sanjana Ramesh, MPH, PhD
(Co-investigator)
Endeavor Health

Gina Menyah
(Co-investigator)
In The Know, Inc.

Shane Reed, MS
(Research Coordinator)
Endeavor Health

Endeavor Health
