# Supplementary material for: Breaking barriers to completing genetic testing for inherited breast cancer among at-risk Black women using a community-based participatory research approach
Source: HGG Adv. 2026 Mar 17;7(2):100591. doi: 10.1016/j.xhgg.2026.100591 (PMC13071439; doi:10.1016/j.xhgg.2026.100591)
Supplement: Document S2. Article plus supplemental information [file mmc2.pdf]

HGG Advances

ARTICLE

Breaking barriers to completing genetic testing for inherited breast cancer among at-risk Black women using a community-based participatory research approach

Sarah H. Choi,[1,5,*] Sanjana Ramesh,[1] Shanequa Reed,[1] Georgina Menyah,[2] Pamela Ganschow,[3] Vida Henderson,[4] and Henry M. Dunnenberger[1]

Summary

National guidelines from the US Preventive Services Task Force and the National Comprehensive Cancer Network recommend the use of family-health-history (FHH)-based risk assessment tools to guide genetic testing (GT) among women with an increased risk of inherited cancer and inform personalized cancer risk management. Prior research has focused on attitudes toward and decisions about initial uptake of GT in Black patients but little is known about the factors that impact the subsequent completion of GT after they have already provided consent. Using a community-based participatory research (CBPR) approach, we aimed to identify barriers and actionable strategies to improve GT completion offered through the Breast Health Assessment (BHA), an FHH screening tool administered at routine mammography visits. We conducted semi-structured interviews with 12 Black women who screened high-risk for inherited breast cancer and consented to GT through the BHA, but did not complete saliva sample collection. Thematic analysis revealed that lack of dedicated support throughout the BHA workflow emerged as a key obstacle to sample collection, whereas medical mistrust, shame, and limited knowledge were largely regarded as cultural barriers that had no impact on GT completion. Low utilization among participants reflected logistical challenges highlighting the need to evaluate multi-level implementation processes to better understand and address inequities in GT completion. Participants suggested implementing early educational outreach, culturally relevant messaging, and interpersonal touchpoints to promote GT uptake. By applying a CBPR approach, we translated these findings into actionable, equity-focused strategies to improve GT completion within a population genetic screening program.

Introduction

National guidelines from the US Preventive Services Task Force and the National Comprehensive Cancer Network (NCCN) recommend the use of family-health-history (FHH)-based risk assessment tools to guide genetic testing (GT) among women with an increased risk of inherited cancer and inform personalized cancer risk management strategies, including enhanced surveillance, targeted therapies, and risk-reducing interventions.[1–5] Routine screening for FHH of cancer is an accessible, cost-effective, and practical strategy for accurate risk assessment and timely identification of individuals eligible for GT, especially when supported by clinical decision support tools.[6,7] Despite recommendations, implementation of systematic FHH screening and subsequent GT remains inconsistent due to workflow, provider, and system-level barriers.[8,9] Innovative implementation strategies are needed to address care gaps and effectively integrate these tools into routine clinical workflows.

In 2018, Endeavor Health introduced an electronic health record (EHR)-integrated FHH screening tool called the Genetic and Wellness Assessment (GWA).[10] Building upon the GWA, the Breast Health Assessment (BHA) was launched in 2021 to primarily assess for an individual's risk for hereditary breast and ovarian cancer syndrome based on the NCCN guidelines[4] and offer GT at the time of routine screening mammogram. Prior work has shown that integrating genetic screening into routine mammography visits can identify high-risk individuals who might otherwise remain undetected, creating new opportunities to reduce morbidity and mortality through evidence-based and tailored cancer risk management.[11] Similarly, the BHA was designed and implemented to streamline risk identification and increase patient access to guideline-recommended GT for women at increased risk of inherited breast cancer (IBC).

Despite the promise of population genetic screening, disparities persist in the utilization of GT services. Research consistently demonstrates that Black women are less likely to complete GT, limiting access to personalized care and potentially widening ongoing inequities in breast cancer outcomes.[1,12–15] Although known barriers to GT in this population include lack of knowledge, decreased access, cost, cultural beliefs, mistrust in health system, and genetic data privacy concerns,[16–20] prior studies have largely focused on attitudes toward and decisions about initial uptake of GT. Little is known about the

[1]Neaman Center for Personalized Medicine, Endeavor Health, Evanston, IL 60201, USA; [2]In the Know, Inc., Chicago, IL 60607, USA; [3]Internal Medicine, University of Illinois Cancer Center, Chicago, IL 60612, USA; [4]Cancer Prevention Program, Public Health Sciences Division, Fred Hutch, Seattle, WA 98109, USA
[5]Lead contact
*Correspondence: sarah.choi@endeavorhealth.org
https://doi.org/10.1016/j.xhgg.2026.100591.
© 2026 The Authors. Published by Elsevier Inc. on behalf of American Society of Human Genetics.
This is an open access article under the CC BY license (http://creativecommons.org/licenses/by/4.0/).

factors that impact the subsequent completion of GT after at-risk Black patients have given consent for GT.

Preliminary data from the BHA revealed disproportionately low rates of GT completion, defined as failure to provide a saliva sample after providing prior electronic consent via the BHA, among Black women who screened high-risk for IBC. This observation suggested a critical but underexplored gap within the BHA clinical workflow. Individuals who agree to GT but do not complete it represent a distinct and actionable population, as barriers at this stage are more likely to reflect modifiable logistical, communication, or system-level factors rather than lack of interest or attitudinal resistance. This group is often invisible in implementation research, yet they represent a meaningful source of downstream inequity when system-level barriers prevent follow-through after initial engagement. Understanding these barriers is critical for increasing follow-through to complete GT and ensuring that population-based genetic screening programs deliver equitable benefits.[21]

To address this gap, we conducted a qualitative study using a community-based participatory research (CBPR) approach to examine the experiences of Black women identified as high-risk through the BHA, who provided electronic consent to guideline-recommended GT but did not complete sample collection at the time of their annual screening mammogram. Our objective was to identify barriers and facilitators specific to this stage of the care pathway and co-develop actionable steps to improve equitable GT uptake within the BHA clinical workflow at our community health system.

Subjects, materials, and methods

Engaging historically and currently underrepresented communities in genomics research requires approaches that are committed to bi-directional learning and shared decision-making.[22,23] Community engagement provides a way in which both researchers and community members can actively and equitably partake in the research process.[22,23] In this study, we employed a CBPR approach, which promotes collaboration among all stakeholders in the development and implementation of research.[24] Examples of how CBPR principles were applied in the study are described in Table 1.[25]

Establishment of CBPR and research team partnership

At project inception, a member of the research team had an established relationship with a trusted community advocate actively engaged in cancer prevention and GT outreach within the Black community. This individual was invited to serve as the community lead for the CBPR partnership and helped in recruiting seven additional members to participate as CBPR team members through relationship built from past community and advocacy events. These eight individuals formed the CBPR team and guided all phases of the study, contributing their lived experiences as Black women with personal and/or family histories of cancer.

CBPR team engagement included a series of collaborative meetings focused on relationship building, mutual education, and study co-design. All members were trained in study methods and helped create recruitment materials, refine interview questions, inform IRB planning, co-interpret interview findings, and contribute to dissemination planning. Their lived experiences ensured that research translation was culturally sensitive, inclusive, and grounded in their community's priorities. To encourage meaningful engagement, CBPR team members were provided accommodations, such as evening meetings, and they received compensation for their time.

BHA workflow

The BHA is sent via NorthShore*Connect* (NSC), our secure patient messaging portal, to all patients with a scheduled screening mammogram to complete before their appointment. Patients who screen positive on the BHA, thereby meeting NCCN criteria for germline GT, are offered to complete GT with a comprehensive hereditary cancer panel, which is typically covered by insurance or through a financial assistance program for patients who meet criteria. After electronic patient consent is provided, oral saliva sample self-collection is offered during the patient's mammogram appointment (Figure 1).

Participant identification and recruitment

From August 2021 to December 2023, 38,895 patients completed the BHA via NSC. Of these, 9,797 (25.19%) screened positive, or were identified as having a high-risk personal or family history for IBC based on the NCCN guidelines, and were recommended and offered a comprehensive hereditary cancer panel for GT. Among those who completed the BHA, 1,974 (5.08%) were documented as Black or African American in the EHR, of whom 548 (27.76%) screened positive. While 160 (29.20%) of these at-risk Black women consented to GT, 68 (42.50%) did not proceed with testing. This preliminary review revealed a low GT completion rate among consented Black patients and was a key factor that motivated our research.

Eligible participants included self-identified Black women who screened high-risk for IBC on the BHA and consented to GT but never followed through with sample collection at their screening mammogram appointment. At the time of recruitment, we identified 81 potential participants through a combination of data extraction from the EHR and manual chart review as not having completed GT by submitting a saliva sample. Across multiple meetings, the research and CBPR teams collaboratively reviewed recruitment materials and procedures, with CBPR members providing input on participant outreach and recontact methods. CBPR

Table 1. Application of CBPR principles in the study

CBPR key principle[25]	Application in the study
(1) Recognizes community as a unit of identity	Community was defined as Black women who screened high-risk for IBC and were recommended to complete GT based on their FHH. CBPR team members shared a collective identity shaped by lived experiences as a Black woman with a personal and/or family history of cancer
(2) Builds on strengths and resources of the community	CBPR team members shared experiential knowledge and cultural insights, which informed recruitment strategies, interview guide development, and interpretation of study findings
(3) Facilitates collaborative partnerships in all phases of the research	CBPR team members engaged in shared decision-making across all phases of the study, including study design, recruitment planning, interview guide development, and data interpretation and dissemination
(4) Integrates knowledge and action for mutual benefit of all partners	Qualitative findings were translated into actionable implementation strategies to improve GT completion among patients engaged in the BHA. CBPR team members gained tools and knowledge to share education about GT within their community networks
(5) Promotes a co-learning and empowering process that attends to social inequalities	Researchers gained insight into community lived experiences, cultural context, and system-level barriers, while CBPR team members developed research literacy and confidence to engage in GT education
(6) Involves a cyclical and iterative process	Recruitment planning and data analysis went through an iterative process, with lessons shared with the CBPR team across multiple meetings to refine strategies, themes, and recommendations based on team's feedback
(7) Addresses health from both positive and ecological perspectives	The study examined not only barriers and facilitators to GT completion but also motivations, and broader system and workflow factors influencing GT follow-through
(8) Disseminates findings and knowledge gained to all partners	Findings were shared with CBPR team, who contributed to interpretation and expressed commitment to disseminating GT information and study insights within their community networks

members directly edited materials to ensure language was clear, culturally appropriate, and acceptable to participants.

Co-developed messages detailing the study purpose, eligibility criteria, and instructions for scheduling an interview were sent to all potential participants via NSC (Figure 2). Patients were asked to contact the study coordinator if interested in participating in the study and were scheduled to complete a one-time qualitative interview via Webex,[26] a HIPAA-compliant videoconferencing platform. Prior to the scheduled interview, participants received an NSC reminder message containing call-in information and the informed consent document. Audio-recorded interviews were conducted after obtaining informed verbal consent from participants, and those who opted to use video were also video-recorded. This study was approved by the Institutional Review Board at Endeavor Health (IRB no. STUDY00000094; approved November 14, 2024).

Data collection and procedures

Semi-structured interviews took place via Webex from January to March 2025. All interviews were conducted by the first and second authors (S.H. Choi and S. Ramesh) and lasted between 35 and 50 min. The interview guide was informed by and co-developed with our CBPR team. A collaborative process was used to develop the interview guide, with CBPR members suggesting question phrasing and probes to promote meaningful discussion.

Participants were asked open-ended questions about their reasons for completing the BHA, consenting to GT, and barriers to providing a sample at their mammogram appointment. Perceived community and cultural norms were also explored to assess whether others' views influenced their attitudes or decisions toward GT (supplemental information, S1). Demographic data were collected at the end of the interview to characterize the study sample. The interviews were audio-recorded, transcribed, and de-identified prior to analysis. Participants received a $50 Visa gift card in compensation for their time.

Data analysis

The audio-recorded, qualitative interviews were professionally transcribed for thematic content analysis.[27] An *a priori* codebook was created based on interview questions that were guided by domains from the Integrated Model of Behavioral Prediction (perceived knowledge, attitudes, norms, self-efficacy, and salience of behavior).[28] The first two authors (S.H.C. and S. Ramesh) re-familiarized themselves with a subset of the data, using *a priori* codes for deductive coding and memoing to generate inductive codes. After testing the initial codebook and establishing high intercoder reliability (Krippendorff's $\alpha = 0.8$),[29] S.H.C. and S. Ramesh independently coded the remaining interviews in small batches, meeting after each round to discuss new codes and refine the codebook using a consensus-based approach. S. Ramesh exported quotes grouped by code frequency and narratively summarized what participants said about each code and code family. Analysts and the research team discussed preliminary findings, which were then shared with the CBPR team in order to finalize themes and make recommendations. This process involved multiple meetings in which

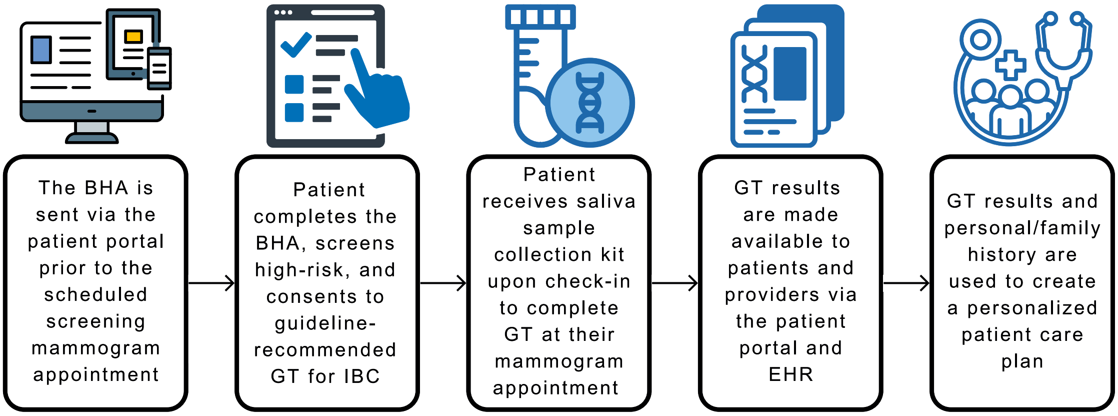

Figure 1. Breast Health Assessment workflow
Workflow of a patient with a scheduled screening mammogram going through the BHA program, from risk identification to genetic testing completion and post-results follow-up.

CBPR members reviewed emerging themes and exemplary quotes and provided feedback on their relevance, cultural context, and resonance with lived experiences. Community participation in data analysis informed actionable solutions, which was an intentional CBPR team-driven step, to break down GT barriers among Black women with increased risk for IBC within the BHA workflow. A community report of study findings was co-developed with the CBPR team and was disseminated to participants who expressed interest in receiving study results (supplemental information, S2).

Results

Participant characteristics

Among the potential participants ($N = 81$) recruited for the study, 12 individuals consented to participate in an interview. All were self-identified Black women who initially provided electronic consent to the recommended GT via the BHA but did not complete saliva sample collection at their screening mammogram appointment. The majority of participants were 45 years or older, had completed at least some college education, had biological children, and worked full time (Table 2).

Interview themes

Three overarching themes were identified, each with three subthemes that captured motivations for GT, barriers and facilitators to completing BHA-recommended GT among at-risk Black women (Table 3), and participant-suggested recommendations for improving GT completion among Black women and the broader African American community both within and beyond the BHA (Table 4).

Participants held positive attitudes and motivations to complete GT

Recognition of GT and its clinical value. Most participants were aware of the clinical benefits of GT for inherited cancer risk before they were recommended through the BHA. Mass media was frequently referenced as an information source: "I've heard about it like on TV, or you hear about a lot of stories where women got the genetic testing and found out that they could be susceptible to all kinds of other cancers or all kinds of other issues" (P02, chose not to submit a sample). Others knew family members and friends who had completed GT after cancer diagnosis: "I've had friends impacted by cancer and … like their family and friends doing [genetic testing] after they've been diagnosed" (P05, sample failure). Despite having a general understanding of its clinical value, participants had never received a GT recommendation until the BHA, including those with a strong family history of cancer. One participant indicated regret for not having received information about its personal utility sooner: "Nobody ever told me how beneficial it could be before now, before this conversation, before I was flagged … Had I known, I probably would've done it sooner" (P08, never received kit).

Desire to complete GT for themselves and their family. Participants expressed strong instrumental attitudes that influenced their GT consent decision, such as a desire for health information for themselves and their families. GT was frequently viewed as providing much deeper insights into breast health than routine clinical exams such as mammograms: "Mammogram is what's here in my breast right now, whereas that genetic is down the line, it could be a preventive measure" (P01, chose not to submit a sample). Furthermore, GT provided participants with an opportunity to be proactive about their health rather than reactive: "It's an information tool that we can all benefit from so that we can put some precautionary things in place" (P10, never received kit). Several emphasized the importance of completing GT to protect future generations: "It helps my family. I can tell my daughters … granddaughters … grandsons down the line that, okay, this is what they kind of figured out's going on in our family line" (P02, chose not to submit

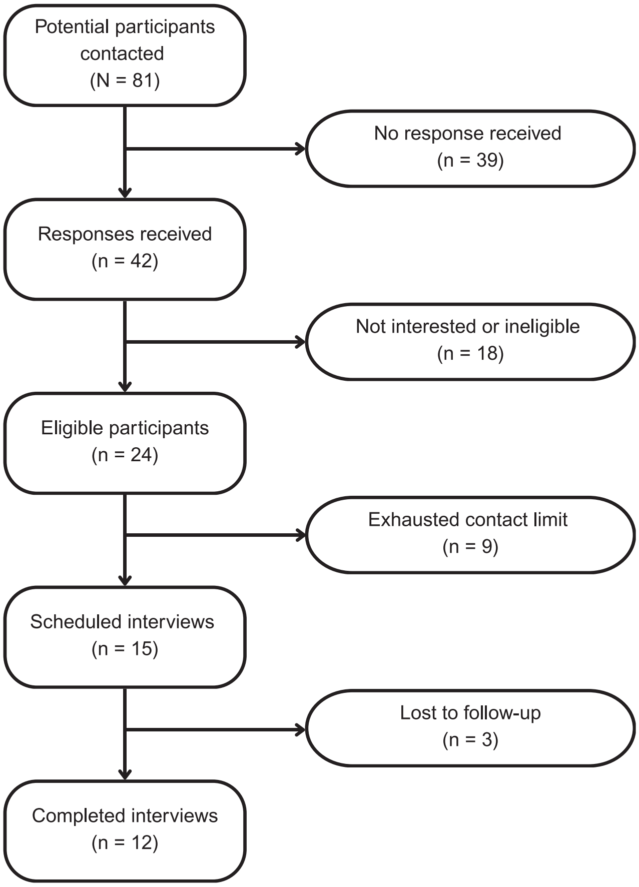

Figure 2. Participant recruitment flowchart
Flowchart of the participant recruitment process for Black women who consented to guideline-recommended genetic testing through the BHA but did not complete sample collection at their screening mammogram appointment.

a sample). One participant even acknowledged its clinical utility for patients without a strong family history: "You could be the first of that family. So, I think it should be something that should be a standard of care as prevention for everyone, regardless of family history" (P06, never received kit).

Minimal privacy and confidentiality concerns. Participants frequently acknowledged cultural barriers to GT in the Black community, including mistrust of the medical system rooted in historical injustices: "Unfortunately, the African American community is not very trusting of the health systems because of the history of doing things on people without permission" (P06, never received kit). Another participant discussed how medical distrust contributes to skepticism and disbelief in the value of GT: "There are lots of barriers attached to anything genetics" (P10, never received kit). However, it is crucial to note that participants did not perceive these sociocultural beliefs as personal barriers and expressed minimal to no concerns that would negatively impact or change their decision to complete GT. As one explicitly stated: "Well, I already know that HIPAA exists … so I don't have any fears of confidentiality" (P09, sample failure). Informed decision-making was also facilitated by trust in the health system and providers: "I just feel confident about it because I know that so many times when I have had tests and went to the doctor … they stressed privacy and confidentiality. So, I feel comfortable" (P07, chose not to submit a sample). Participants were not worried about the testing laboratory nor sample mishandling: "I do trust my healthcare system, so I was hoping that if it was something that they were working with, then I would be okay" (P05, sample failure).

BHA logistical issues primarily contributed to GT incompletion

Gaps in BHA clinical workflow. Despite consenting to GT, half of our participants ($n = 6$) were never given a saliva sample collection kit upon arrival. Most were unaware that they were supposed to receive a kit from the medical receptionist: "I really didn't know that that was an option at the time of my visit" (P06, never received kit). Those who expected the kit were frustrated by the lack of follow through: "If I'm signing up for something and I'm providing you the information you need in order for you to then give me what it is I'm agreeing to, then do your due diligence to follow through to provide me with what I need" (P12, never received kit). These participants often described how they would have completed GT "at the drop of a dime" (P10, never received kit) had they been handed the kit, as intended.

Lack of support and follow-up throughout BHA process. - Three of the six people who received a sample collection kit later learned that the sample had failed. One participant recalled receiving minimal support from the nurse who was on staff: "She wasn't helpful. She just gave me the test and told me to follow the instructions" (P04, sample failure). This participant also expressed low perceived confidence to resubmit their new sample by mail: "I never used the other kit because I read the directions again and I didn't want to do it wrong again" (P04, sample failure). The other two who had sample failure reported that family and personal health issues kept them from resubmitting their mail-in GT kit. Of the remaining three who chose not to submit a saliva sample, all still had an interest in completing GT, but were overwhelmed during their mammogram appointment and did not view it as an immediate priority: "I didn't even open it … I thought to myself I just want to get out of here right now, and I'll do it later, and later just never came" (P01, chose not to submit a sample). These participants also highlighted the importance of timely follow-up for those who did not complete testing at their appointment.

Screening mammogram is convenient for GT. Although participants cited several workflow issues that hindered sample collection, they generally agreed that screening mammograms were a valuable opportunity to increase GT access and uptake: "I think that was an effective way of doing it. I wish more people; more women had the opportunity to give the sample. I hate that I ruined my

Table 2. Participant characteristics ($N = 12$)

	N	(%)
**Age (years)**		
35–44	1	(8)
45–54	4	(33)
55–64	5	(42)
Over 65	2	(17)
**Highest education level**		
High school	1	(8)
Some college (or certificate)	5	(42)
College graduate	2	(17)
Post-graduate degree (MA, MS, PhD, MD, DO, etc.)	4	(33)
**Annual household income**		
Less than $25,000	2	(17)
$25,000–$49,999	1	(8)
$50,000–$99,999	3	(25)
$100,000–$199,999	4	(33)
$200,000–$299,999	1	(8)
$300,000 or above	1	(8)
**Employment status**		
Full time	7	(58)
Part time	1	(8)
Student	1	(8)
Disabled	1	(8)
Retired	2	(17)
**Current relationship status**		
Married	5	(42)
Single	4	(33)
Separated or divorced	2	(17)
Widowed	1	(8)
**Biological children**		
Yes	8	(67)
No	4	(33)
**Religion**		
Protestant	7	(58)
None	3	(25)
Other	2	(17)

sample. But yeah, I think it's a good thing" (P09, sample failure). Participants expressed support for offering GT at the same time as their screening mammogram to save time: "That would be perfect, because that's not something that I need to walk out the door with, that's not something that I would have to do at home, I could do it right there, in that moment, and it's taken care of" (P08, never received kit). Another participant went on to share: "I think it would be really convenient for busy individuals, mothers, let's put it all in one appointment" (P02, chose not to submit a sample). Although participants viewed GT at screening mammograms favorably, they emphasized the need for clear information and expectations throughout the entire process.

Table 3. Interview themes and exemplary quotes

Main theme	Subthemes	Exemplary quotes
Participants held positive attitudes that motivated GT decision-making	(1) Prior recognition of GT and its clinical value	"I've heard about genetic testing. I personally think it's awesome" (P02, chose not to submit a sample)
	(2) Desire to complete GT for themselves and their family	"It does benefit everyone. Then if I find … I am at risk, then I can let my family members know" (P06, never received kit)
	(3) No privacy and confidentiality concerns	"I guess it really doesn't matter. When I get my blood work from my doctor, I don't ask what lab it's being done by. You know what I mean? So it's not a concern" (P07, chose not to submit a sample)
BHA logistical issues primarily contributed to GT incompletion	(1) Gaps in BHA clinical workflow	"I didn't know I was supposed to get a saliva kit or whatever. So to be fair, nobody really followed up with me. It was just we were all just focused on getting the mammogram done" (P03, never received kit)
	(2) Lack of support and follow-up throughout BHA process	"So I did pick up the test, but I did not complete the testing … it was just recommended to me that if I wanted to get genetic testing … It wasn't recommended as in you really need this" (P07, chose not to submit a sample)
	(3) Screening mammogram is convenient for GT	"I think one of the biggest things for me is that, and I'll say it again, convenient, that my [hospital], they're providing this, and I don't have to kind of go to a third-party, I think that's a big benefit for me" (P08, never received kit)

Participant-suggested recommendations for improving GT completion among at-risk Black women

Start the conversation early. Participants perceived that information about genetics and GT is not widely shared in the community, and that healthcare providers frequently fail to discuss these topics with them, leaving them feeling uninformed. They discussed significant gaps in awareness: "There's not enough context around it, I don't think that it is talked about a lot, I don't think that the information is shared" (P08, never received kit). As one participant noted: "Lack of information leads to the fear and to the shame" (P02, chose not to submit a sample). Another participant emphasized how these emotional responses may further impede information seeking about GT:

> "They will chalk it up to, 'I'm not going to understand it,' 'I'm not going to know what they are talking about.' They don't want to ask questions because they may be embarrassed to show or feel like they don't know what they're talking about" (P12, never received kit).

Increase community-based outreach and engagement. Participants discussed how a lack of trusted advocates in their community may contribute to reluctance or hesitancy toward GT: "I just think we just need to have better advocates for our health and I think once that happens, then we'll listen more (P06, never received kit). They highlighted the importance of raising awareness through ongoing community engagement and consistent communication, such as signs, brochures, and educational classes. One participant mentioned that community engagement would be more effective if led by someone from their own community:

> "I'm going to say, have [somebody who is] an African American to help reach out to the community on behalf of y'all. Not saying that like other folks wouldn't be received, but it just hits different when it comes from somebody from your own community" (P03, never received kit).

Integrate interpersonal touchpoints for human connection. In addition to community outreach, participants expressed a strong desire for an interpersonal touchpoint with trusted healthcare providers and community advocates to bridge information gaps and build trust: "Sometimes you just want to hear, 'Hey, this is just to help you in case there is something, then the doctors can tackle it early.' Just providing that emotional support sometimes goes a long way" (P07, chose not to submit a sample). Many suggested having dedicated support from staff with genetics expertise to provide one-on-one guidance when involving patients in the GT process:

> "Going back to the whole embarrassment kind of thing, sometimes we lose, or we have lost also the human interaction. And for some African-Americans too, they're not all tech-savvy. And so the one-on-one is still very important" (P12, never received kit).

Several participants agreed that information about GT would be most effective if delivered by trusted healthcare providers, such as their primary care physicians:

> "Maybe the person that communicates this and that offers this level of testing should be their primary care doctor, people that they have regular communication because there's a trust factor that's there. And that's been one of the barriers to us receiving a lot of care in the past, and probably now, is those concerns" (P10, never received kit).

Discussion

This qualitative study addresses a critical but underexplored gap in the delivery of GT through an FHH screening tool (i.e., BHA) by examining why some Black

Table 4. Participant-suggested recommendations for improving GT uptake among at-risk Black women

Recommendation	Exemplary quotes
Start the conversation early: implement early education and regular communication to promote GT	"Information should be available at mammogram appointments, gynecologist appointments, other appointments, even internal medicine, when people go to their gen for checkups and things like that … the providers mentioning it and talking about it, providing a brochure. Digital information too" (P05, sample failure)
	"I would say educate them, give them the pros and the cons of doing it, not doing it. Again, it's a matter of life and death. Early detection is the key to any illness. Early, I just say educate" (P04, sample failure)
	"The more glossy handouts you can put together. The more information, the more communication. If more people know about it and the advantages of it, and get a little bit more of the background on how it's taken, and analyzed, and put together, and how the information is then transcribed or given to us, I think there would be more opportunities for more women to sign up for genetic testing" (P08, never received kit)
Increase community outreach and engagement: build and utilize champions from within the community	"Just get the information out [in the community]. Grocery stores would be helpful, and community centers" (P11, never received kit)
	"Offering some type of class, or seminar explaining the importance of the genetic testing. Maybe if once a month Endeavor had some kind of program where it talked about why that's important" (P12, never received kit)
	"Have [somebody who is] an African American to help reach out to the community on behalf of y'all. Not saying that like other folks wouldn't be received, but it just hits different when it comes from somebody from your own community" (P03, never received kit)
Add interpersonal touchpoint for human connection: include opportunities for one-on-one guidance from trusted providers	"For some African-Americans, one-on-one is still very important. And if they get [dedicated support] there, when they're checking in for their mammogram, there's that human interaction but there's that personal interest too. And that might spur them to take that initiative to [participate]" (P12, never received kit)
	"I really do think it would've been helpful to have a provider input at that time. I would say the nurse because I'm not sure if the technician would know" (P04, sample failure)
	"Maybe the person that communicates this and that offers this level of testing should be their primary care doctor, people that they have regular communication because there's a trust factor that's there" (P10, never received kit)

women who initially consent to guideline-recommended GT do not complete testing within our community health system. We identified key barriers and facilitators to post-consent GT completion as well as broader participant-suggested considerations to improve follow-through among Black women identified as high-risk for IBC. Aligned with CBPR principles,[25] we meaningfully translated qualitative findings into actionable strategies that target an understudied post-consent step in the implementation process.[16] Employing CBPR created mutual benefits for both the research and community team members, empowering community members as advocates who expressed commitment to disseminating GT information within their own social communities.

Our findings show that lack of GT completion among study participants was primarily due to logistical challenges, including not receiving the saliva sample collection kit or lacking follow-up support, rather than historical or sociocultural factors that are often discussed in the literature.[15,16,19,20] While participants acknowledged persisting sociocultural barriers in their communities, including historical mistrust of the medical system and skepticism about GT, these factors were not perceived as personal barriers and did not strongly influence their initial decision to consent or subsequent decisions to complete testing. This contrasts with most prior studies, which report that cultural barriers within the Black community strongly influence decisions to undergo GT for inherited cancer risk.[15,20,30–33] The absence of strong sociocultural barriers in a late post-consent stage does not negate their importance earlier in the care pathway but rather suggests that passive system failures may disproportionately affect Black women who are otherwise motivated to proceed. Accordingly, our results highlight the need to evaluate multi-level implementation processes within the BHA clinical workflow to identify and address post-consent gaps in GT and improve follow-through with sample collection.

Participants provided practical recommendations to encourage GT completion among Black women at-risk for IBC. These included prioritizing early education, consistent communication, and increasing community awareness through culturally accessible strategies such as signage, brochures, and seminars delivered in clinics, grocery stores, and community centers. Many of these strategies have been described previously, emphasizing the need for culturally tailored or sensitive approaches when engaging marginalized communities.[17,30,31,33–35] Our findings suggest that intervention efforts must also be culturally relevant and reflect the lived experiences of the community to promote equitable engagement.

An important point shared by both participants and CBPR team members was that human connection and interpersonal touchpoints are crucial to engaging Black women in GT as well as in health care in general. Prior research has shown that, in addition to insufficient knowledge and awareness, when care feels impersonal, Black patients report greater mistrust, disengagement, and skepticism toward medical recommendations, including GT.[15,30,32,33] While many studies suggest

intervention strategies such as patient and community education, provider training, expanded insurance coverage, and scalable and systematic approaches to reduce disparities in the use of GT,[16,30,31,36] our results highlight the importance of having a "warm hand-off" to support patient navigation, foster trust and confidence, and provide clarity during the multistep GT process. Such interpersonal connection not only addresses cultural barriers in the broader Black community but also can help overcome logistical challenges identified in the clinical workflow. As health systems increasingly rely on automated, scalable approaches for implementing risk assessment tools, our results raise an important question: How much automation is too much? Personalized support may remain crucial for promoting equitable GT uptake.

Limitations of the study

Due to the relatively small sample size of the study, our findings may not be generalizable to the broader Black community or to individuals receiving care outside our health system. Participants' lived experiences and perspectives represent perceived barriers and facilitators within this context and may not reflect all experiences of Black women across population genetic screening programs. This pilot study focused specifically on the experiences of patients who consented to but did not complete GT within a unique population genetic screening program (i.e., the BHA) at our institution, with the goal to identify implementation strategies in improving GT completion. In addition, participation in the study may have been limited by potential technology barriers as all communication and interview participation involved access to the electronic patient portal and a virtual videoconferencing platform. This limitation may be minimal, given that the BHA program was also delivered electronically. It is also important to recognize that recall bias may have influenced participant responses, as some interviews were conducted months to over a year after their mammogram appointment. Lastly, participants had already completed the BHA and consented to GT, which may reflect a more engaged group with more positive views toward GT than those who did not engage at all.

Conclusion

This qualitative study identified key barriers and facilitators to GT completion after consent among Black women at increased risk for IBC, identified through a FHH screening tool (i.e., BHA) within our community health system. Although participants perceived cultural mistrust and limited knowledge as persistent barriers within the broader community, post-consent GT incompletion was largely influenced by logistical and system-level failures, including workflow errors and lack of dedicated follow-up support. These findings suggest that, once consent is provided, incompletion of testing reflects gaps in care delivery rather than lack of interest or attitudinal resistance. Applying a CBPR approach allowed us to co-develop actionable strategies aimed to improve post-consent GT completion among Black women with increased risk of IBC. Future efforts should focus on integrating human touchpoints into scalable clinical workflows, expanding culturally tailored outreach, and engaging in community partnerships to support GT completion and follow-through.

Data and code availability

There are restrictions to the availability of our qualitative data due to the possibility that identifying information is present in participant responses. De-identified data and study materials presented in this paper may be made available by the corresponding author upon request.

Consortia

The community members of the Community-Based Participatory Research Team are Latonya Carter, Tami Daniel, Ontisar Freelain, Peggy Gayle, Ama Johnson, Michelle McKenith, and Turqueya Wilson.

Acknowledgments

We thank the mammography clinic patients for their participation in this study. We also acknowledge Christopher Ward for their valuable contribution to this project. Funding for this study was provided by the Institute for Translational Medicine.

Author contributions

Conceptualization, S.H.C., P.G., V.H., and H.M.D.; formal analysis and methodology, S.H.C., S. Ramesh, S. Reed, and G.M.; funding acquisition, S.H.C. and H.M.D.; investigation, S.H.C., S. Ramesh, and G.M.; validation, S.H.C., S. Ramesh, S. Reed, G.M., P.G., V.H., and H.M.D.; writing – original draft, S.H.C., S. Ramesh, S. Reed; writing – review & editing, S.H.C., S. Ramesh, S. Reed, G.M., P.G., V.H., and H.M.D.

Declaration of interests

G.M. has received compensation from Endeavor Health for their consulting work on this project.

Supplemental information

Supplemental information can be found online at https://doi.org/10.1016/j.xhgg.2026.100591.

Received: November 26, 2025
Accepted: March 12, 2026

References

1. Cragun, D., Weidner, A., Lewis, C., Bonner, D., Kim, J., Vadaparampil, S.T., and Pal, T. (2017). Racial disparities in *BRCA* testing and cancer risk management across a population-based sample of young breast cancer survivors. Cancer *123*, 2497–2505. https://doi.org/10.1002/cncr.30621.

2. Childers, C.P., Childers, K.K., Maggard-Gibbons, M., and Macinko, J. (2017). National Estimates of Genetic Testing in Women With a History of Breast or Ovarian Cancer. J. Clin. Oncol. *35*, 3800–3806. https://doi.org/10.1200/JCO.2017.73.6314.
3. Monticciolo, D.L., Newell, M.S., Moy, L., Niell, B., Monsees, B., and Sickles, E.A. (2018). Breast Cancer Screening in Women at Higher-Than-Average Risk: Recommendations From the ACR. J. Am. Coll. Radiol. *15*, 408–414. https://doi.org/10.1016/j.jacr.2017.11.034.
4. National Comprehensive Cancer Network (2025). Genetic/Familial High-Risk Assessment: Breast, Ovarian, and Pancreatic V.1.2025. https://www.nccn.org/guidelines.
5. U.S. Preventive Services Task Force (2025). BRCA-Related Cancer: Risk Assessment, Genetic Counseling, and Genetic Testing. https://www.uspreventiveservicestaskforce.org/uspstf/recommendation/brca-related-cancer-risk-assessment-genetic-counseling-and-genetic-testing.
6. Orlando, L.A., Wu, R.R., Myers, R.A., Neuner, J., McCarty, C., Haller, I.V., Harry, M., Fulda, K.G., Dimmock, D., Rakhra-Burris, T., et al. (2020). At the intersection of precision medicine and population health: an implementation-effectiveness study of family health history based systematic risk assessment in primary care. BMC Health Serv. Res. *20*, 1015. https://doi.org/10.1186/s12913-020-05868-1.
7. Wu, R.R., Myers, R.A., Neuner, J., McCarty, C., Haller, I.V., Harry, M., Fulda, K.G., Dimmock, D., Rakhra-Burris, T., Buchanan, A., et al. (2022). Implementation-effectiveness trial of systematic family health history based risk assessment and impact on clinical disease prevention and surveillance activities. BMC Health Serv. Res. *22*, 1486. https://doi.org/10.1186/s12913-022-08879-2.
8. Paul, J.L., Leslie, H., Trainer, A.H., and Gaff, C. (2018). A theory-informed systematic review of clinicians' genetic testing practices. Eur. J. Hum. Genet. *26*, 1401–1416. https://doi.org/10.1038/s41431-018-0190-7.
9. Schaibley, V.M., Ramos, I.N., Woosley, R.L., Curry, S., Hays, S., and Ramos, K.S. (2022). Limited Genomics Training Among Physicians Remains a Barrier to Genomics-Based Implementation of Precision Medicine. Front. Med. *9*, 757212. https://doi.org/10.3389/fmed.2022.757212.
10. David, S.P., Dunnenberger, H.M., Choi, S., DePersia, A., Ilbawi, N., Ward, C., Wake, D.T., Khandekar, J.D., Shannon, Y., Hughes, K., et al. (2023). Personalized medicine in a community health system: the NorthShore experience. Front. Genet. *14*, 1308738. https://doi.org/10.3389/fgene.2023.1308738.
11. Brabender, D., Siegel, E., Culver, J.O., Comeaux, J.G., Date, A., and Woodworth, A. (2025). Identification of Patients at Elevated Cancer Risk through a Community-Based Genetic Testing Program. Ann. Surg Oncol. *32*, 9041–9049. https://doi.org/10.1245/s10434-025-17820-w.
12. Reid, S., Cadiz, S., and Pal, T. (2020). Disparities in Genetic Testing and Care Among Black Women with Hereditary Breast Cancer. Curr. Breast Cancer Rep. *12*, 125–131. https://doi.org/10.1007/s12609-020-00364-1.
13. DeSantis, C.E., Ma, J., Gaudet, M.M., Newman, L.A., Miller, K.D., Goding Sauer, A., Jemal, A., and Siegel, R.L. (2019). Breast cancer statistics, 2019. CA Cancer J. Clin. *69*, 438–451. https://doi.org/10.3322/caac.21583.
14. Chapman-Davis, E., Zhou, Z.N., Fields, J.C., Frey, M.K., Jordan, B., Sapra, K.J., Chatterjee-Paer, S., Carlson, A.D., and Holcomb, K.M. (2021). Racial and Ethnic Disparities in Genetic Testing at a Hereditary Breast and Ovarian Cancer Center. J. Gen. Intern. Med. *36*, 35–42. https://doi.org/10.1007/s11606-020-06064-x.
15. Hann, K.E.J., Freeman, M., Fraser, L., Waller, J., Sanderson, S.C., Rahman, B., Side, L., Gessler, S., Lanceley, A., and PROMISE study team (2017). Awareness, knowledge, perceptions, and attitudes towards genetic testing for cancer risk among ethnic minority groups: a systematic review. BMC Public Health *17*, 503. https://doi.org/10.1186/s12889-017-4375-8.
16. Shen, E.C., Srinivasan, S., Passero, L.E., Allen, C.G., Dixon, M., Foss, K., Halliburton, B., Milko, L.V., Smit, A.K., Carlson, R., and Roberts, M.C. (2022). Barriers and Facilitators for Population Genetic Screening in Healthy Populations: A Systematic Review. Front. Genet. *13*, 865384. https://doi.org/10.3389/fgene.2022.865384.
17. Adams, I., Christopher, J., Williams, K.P., and Sheppard, V.B. (2015). What Black Women Know and Want to Know About Counseling and Testing for BRCA1/2. J. Cancer Educ. *30*, 344–352. https://doi.org/10.1007/s13187-014-0740-9.
18. De Marco, M., Cykert, S., Coad, N., Doost, K., Schaal, J., White, B., Young, D., Isler, M.R., and Corbie-Smith, G. (2010). Views on Personalized Medicine: Do the Attitudes of African American and White Prescription Drug Consumers Differ. Public Health Genom. *13*, 276–283. https://doi.org/10.1159/000242199.
19. Catz, D.S., Green, N.S., Tobin, J.N., Lloyd-Puryear, M.A., Kyler, P., Umemoto, A., Cernoch, J., Brown, R., and Wolman, F. (2005). Attitudes about Genetics in Underserved, Culturally Diverse Populations. Community Genet. *8*, 161–172. https://doi.org/10.1159/000086759.
20. Iltis, A.S., Rolf, L., Yaeger, L., Goodman, M.S., and DuBois, J.M. (2023). Attitudes and beliefs regarding race-targeted genetic testing of Black people: A systematic review. J. Genet. Couns. *32*, 435–461. https://doi.org/10.1002/jgc4.1653.
21. Rao, N.D., Kaganovsky, J., Fullerton, S.M., Chen, A.T., and Shirts, B.H. (2023). Factors Influencing Genetic Screening Enrollment among a Diverse, Community-Ascertained Cohort. Public Health Genom. *26*, 113–122. https://doi.org/10.1159/000531989.
22. Lemke, A.A., Esplin, E.D., Goldenberg, A.J., Gonzaga-Jauregui, C., Hanchard, N.A., Harris-Wai, J., Ideozu, J.E., Isasi, R., Landstrom, A.P., Prince, A.E., et al. (2022). Addressing underrepresentation in genomics research through community engagement. Am. J. Hum. Genet. *109*, 1563–1571. https://doi.org/10.1016/j.ajhg.2022.08.005.
23. Wand, H., Martschenko, D.O., Smitherman, A., Michelson, S., Pun, T., Witte, J.S., Scott, S.A., Cho, M.K., Ashley, E.A.; and Preventive Genomics Program Co-Design Working Group (2023). Re-envisioning community genetics: community empowerment in preventive genomics. J. Community Genet. *14*, 459–469. https://doi.org/10.1007/s12687-023-00638-y.
24. Wallerstein, N., and Duran, B. (2010). Community-Based Participatory Research Contributions to Intervention Research: The Intersection of Science and Practice to Improve Health Equity. Am. J. Public Health *100*, S40–S46. https://doi.org/10.2105/AJPH.2009.184036.
25. Israel, B., Schulz, A., and Becker, A. (1998). Review of Community-Based Research: Assessing Partnership Approaches to Improve. Public Health *19*, 173–202.
26. (2025). Webex Meetings. https://www.webex.com/.

27. Hsieh, H.F., and Shannon, S.E. (2005). Three Approaches to Qualitative Content Analysis. Qual. Health Res. *15*, 1277–1288. https://doi.org/10.1177/1049732305276687.
28. Fishbein, M. (2009). An Integrative Model for Behavioral Prediction and Its Application to Health Promotion. In Emerging Theories in Health Promotion Practice and Research, 2nd ed., R.J. DiClemente, R. Crosby, and M.C. Kegler, eds. (Jossey-Bass/Wiley), pp. 215–234.
29. Krippendorff, K. (2019). Content Analysis: An Introduction to its Methodology, 4th ed. (Sage).
30. Rosas, L.G., Nasrallah, C., Park, V.T., Vasquez, J.J., Duron, Y., Garrick, O., Hattin, R., Cho, M., David, S.P., Evans, J., et al. (2020). Perspectives on Precision Health Among Racial/Ethnic Minority Communities and the Physicians That Serve Them. Ethn. Dis. *30*, 137–148. https://doi.org/10.18865/ed.30.S1.137.
31. Rodriguez, N.J., Ricker, C., Stoffel, E.M., and Syngal, S. (2023). Barriers and Facilitators to Genetic Education, Risk Assessment, and Testing: Considerations on Advancing Equitable Genetics Care. Gastroenterology *164*, 5–8. https://doi.org/10.1053/j.gastro.2022.11.021.
32. Sheppard, V.B., and Tercyak, K.P. (2013). Medical Mistrust and Self-efficacy Influence Black Women's Level of Engagement in BRCA1/2 Genetic Counseling and Testing. J. Natl. Med. Assoc. *105*, 17–22.
33. Antony, M., Putnam, E., Peltzer, C., and Levy, A. (2024). A Scoping Review of Medical Mistrust Among Racial, Ethnic, and Gender Minorities With Breast and Ovarian Cancer. Cureus *16*, e62410. https://doi.org/10.7759/cureus.62410.
34. Henderson, V., Chukwudozie, I.B., Comer-Hagans, D., Coffey, V., Grumbach, G., Spencer, S., Rodgers, C., Kaur, R., Newsome, J., Balay, L., et al. (2021). Development of a culturally sensitive narrative intervention to promote genetic counseling among African American women at risk for hereditary breast cancer. Cancer *127*, 2535–2544. https://doi.org/10.1002/cncr.33525.
35. Henderson, V., Madrigal, J.M., Kendall, L.C., Parekh, P., Newsome, J., Chukwudozie, I.B., Comer-Hagans, D.L., Coffey, V., Grumbach, G., Spencer, S., et al. (2022). Pilot study of a culturally sensitive intervention to promote genetic counseling for breast cancer risk. BMC Health Serv. Res. *22*, 826. https://doi.org/10.1186/s12913-022-08193-x.
36. Guan, Y., Barge, H., Escoffery, C., Cellai, M., Alfonso, S., and Johnson, T.M. (2025). Examining barriers and facilitators to implementing evidence-based genetic risk-stratified breast cancer screening in primary care. Front. Cancer Control Soc. *3*, 1521486. https://doi.org/10.3389/fcacs.2025.1521486.

HGGA, Volume 7

Supplemental information

Breaking barriers to completing genetic testing for inherited breast cancer among at-risk Black women using a community-based participatory research approach

Sarah H. Choi, Sanjana Ramesh, Shanequa Reed, Georgina Menyah, Pamela Ganschow, Vida Henderson, and Henry M. Dunnenberger

Supplemental Information Table of Contents

1. **Material S1 Semi-Structured Interview Guide**
2. **Material S2 Community Report of Study Findings**

Material S1 Semi-Structured Interview Guide

Introduction and Informed Consent:

Hello, my name is ________. I am a researcher with the Personalized Medicine team at Endeavor Health. I am calling about your scheduled interview for our research study, “Barriers and Facilitators to Genetic Testing in African American Women at Increased Risk for Inherited Breast Cancer.” Is this time still good for you?

Thank you for agreeing to chat with me today. I appreciate your time and experience on this matter. The interview can last up to 60 minutes and I will be recording our session so I may go back and listen to it. Do you have any questions for me before we begin? (Wait for questions and answer as appropriate)

Before we begin, I would like to review the consent documents we provided you when we scheduled your call. [**review verbal informed consent document**].

[IF patient consents to continue on]

I’m going to start recording the audio so that we have a transcript of our conversation.

[Turn on recorder]

This is [interviewer name], and I am speaking with [participant’s name]. When we create the transcription, we will replace your name with the code [participant ID#].

Do I have your permission to record this interview?

[Record response and begin the interview]

Overview of Interview:

To begin, I will explain a little bit about what we hope to accomplish today. During your last mammogram appointment, you completed an online questionnaire called the Breast Health Assessment (BHA), asking about your personal and family history of cancer. Based on your answers, you were recommended to get genetic testing to determine if you are at higher risk for inherited breast cancer. The goal of this interview is for us to learn more about your thoughts and feelings on the BHA and completing genetic testing at the time of your mammogram screening. We will start with very general questions and work on more specific questions. The most important thing to remember is that there is no single correct answer to our questions. We are genuinely interested in your thoughts and opinions about the BHA and completing genetic testing for inherited breast cancer. You are not required to answer any question you are uncomfortable with and your answers will not affect your healthcare in any way. Please feel free to share information as it comes to mind. Before we get started, do you have any questions for me?

Construct	Question:	Probes:	Notes:
**Knowledge**	What do you remember from the BHA family health history questionnaire?	Is there anything else?	
	Why did you decide to complete the BHA?<br><br>Did you find the questions easy or difficult to answer?<br>Did you feel comfortable answering the BHA questions? Why?	Can you think of anything else?<br>Can you share more about who in your family was diagnosed with cancer?	
	How did you feel after learning that you may have a higher-than-average risk of developing certain types of cancer, such as breast cancer?	Does anything else come to mind?	
	Have you ever been recommended to complete genetic testing before the BHA?	Has anyone ever mentioned genetic testing in your circle (family, friends, siblings, or anyone in your social circle)?	
	What comes to mind when you think about genetic testing services?	Don’t worry, there is no right or wrong answer. Is there anything else that comes to mind?	
	How do you think genetic testing differs from other clinical services, such as mammograms?		
	Before today, what have you heard about genetic testing for inherited cancers such as breast cancer?	Is there anything else you have heard?	

Describe genetic testing for IBC for the participant: Thank you for sharing that information. For the purpose of this study, I am going to give you a little bit of background information about genetic testing for inherited breast cancer and why genetic testing is recommended for women with a personal and/or family history of cancer. If you have a family history of breast or other cancers, especially if there are young diagnoses (e.g., diagnosed before age 50) and multiple cases or a unique pattern of cancers on one side of your family, it is recommended that you get genetic testing to assess your risk for inherited cancer. This testing looks for changes or mutations in your genes that might increase your likelihood of developing certain cancers, including breast cancer. People identified as having a higher than average risk for developing inherited cancer on the BHA are offered genetic testing for genes related to common inherited cancers. If they decide to get genetic testing, they are given a saliva sample collection kit during their mammogram appointment to complete genetic testing.

**Attitudes**	What are your initial thoughts about IBC?<br><br>What types of emotions do you have about IBC?<br><br>What comes up when you think about genetic testing for IBC?	What would be positive about genetic testing for breast cancer risk?<br><br>What concerns do you have about genetic testing for breast cancer risk?	Follow-up on positive and negative thoughts.
	How did you feel about the idea of completing genetic testing (i.e., providing a saliva sample) at the time of your screening mammogram?	Do you have any other thoughts or feelings?	
	How do you think completing genetic testing for inherited breast cancer might benefit you?	What other benefits can you think of?	Is there anyone else?
	What about your family members? Your community?		

	What do you think are the disadvantages or negative effects of genetic testing for inherited breast cancer?<br><br>Can you think of any other concerns?	Additional prompts –<br>- Timing of completing genetic testing<br>- Sample provision<br>- Privacy/Confidentiality<br>- Fear of receiving results	
	What would be important for you to know before you agree to provide a saliva sample for genetic testing?	Additional prompts –<br>- The testing laboratory<br>- The cost (insurance copay)<br>- The risk (EHR documentation, info shared with PCP)	

**Perceived Norms**	How do you think other people of African Descent feel about genetic testing?	How much do their opinions matter to you?	
		On a scale of 1 – 10, with 1 being, their opinions don't matter at all to a 10 being their opinions matter a lot, what would you say?	Why did you pick a [INSERT NUMBER]?
	How do you think your close friends and family would feel about you completing genetic testing for inherited breast cancer?<br><br>Would you inform family, friends, or children of testing?	How much do their opinions matter to you?	
		On a scale of 1 – 10, with 1 being, their opinions don't matter at all to a 10 being their opinions matter a lot, what would you say?	Why did you pick a [INSERT NUMBER]?
	Who do you believe would support your decision to complete genetic testing for inherited breast cancer risk?	What about your family and friends?<br>What is your relationship with this	Why do you believe that is?<br><br>How much do their

		person/people?<br>What about in your community?	opinions matter to you?
	Do you believe anyone would be against your decision to complete genetic testing for inherited breast cancer?	What about your family and friends?<br>What is your relationship with this person/people?<br>What about in your community?	Why do you believe that is?<br><br>How much do their opinions matter to you?
	Whose opinion might influence your decision to complete genetic testing?	What about your healthcare providers? Which one of your healthcare providers? Any organizations?	Why do these people's opinions matter to you?
	Do you know anyone who has completed genetic testing for cancer or other conditions?	Is it important that you know others who have completed genetic testing? Why or why not?	

**Self-Efficacy**	Why did you decide not to complete genetic testing at the time of your screening mammogram after filling out the BHA?<br><br>What made it difficult for you?<br><br>What concerns and/or emotions did you have when you made the decision not to complete testing during the mammogram visit?	Additional prompts – ask participants about:<br>- More time<br>- More information<br>- Healthcare provider input<br>- Presence of support person<br>- Sample collection process	

	What could have helped you to complete genetic testing at the time of your screening mammogram?	Additional prompts – ask participants about:<br>- Timing the sample provision<br>- Information<br>- Healthcare provider input – if so, how?	How?

**Salience of the behavior**	After our discussion today, how important is it to you to complete genetic testing for inherited breast cancer?	On a scale of 1 – 10, with 1 being not important at all to a 10 being very important, what would you say?	Would you like more information on how to complete genetic testing?
	How important do you believe it is for other African American women to complete genetic testing for inherited breast cancer? What about those who have been identified as high-risk?	On a scale of 1 – 10, with 1 being not important at all to a 10 being very important, what would you say?	Can you explain why you believe this?
Thank you so much for sharing your input throughout this interview. Your responses will help us figure out ways to break the barriers that women of African descent experience in accessing genetic testing services. It is important to us that we don't contribute to health inequities in this space. Before we go over your demographic information, we have one last question for you.			
**Wrap-up questions**	What advice do you have that might help us support other women of African descent women identified as high risk to complete genetic testing for inherited breast cancer?	Do you have any last comments or thoughts before we conclude the interview?	

Great, we really appreciate your participation in this study. Now, we will collect your demographic information. Your responses to these questions will be aggregated with the responses of other participants so that we can classify our sample.

1. What is your current age?

   a. 18-24
   b. 25-34

c. 35-44
d. 45-54
e. 55-64
f. Over 65

2. What gender do you identify with?
a. Male
b. Female
c. Both male and female
d. Neither male nor female
e. Transgender male
f. Transgender female

3. How do you describe your ethnicity?
a. Hispanic/Latino
b. Not Hispanic/Latino

4. How would you describe your racial background? (check all that apply)
a. African American/Black
b. Asian
c. American Indian/Alaska Native
d. Native Hawaiian/Pacific Islander
e. White/Caucasian
f. Other

5. What is your employment status?
a. Full time
b. Part time
c. Student
d. Retired
e. Homemaker
f. Looking for employment
g. Not employed and not looking for employment
h. Disabled

6. What is your current household income?

a. Less than $25,000
b. $25,000 to $49,999
c. $50,000 to $74,999

d. $75,000 to $99,999
e. $100,000 to $149,999
f. $150,000 to $199,999
g. $200,000 to $249,999
h. $250,000 to $299,999
i. $300,000 or above

7. What is your current relationship status?

a. Married/Domestic Partner/Civil Union
b. Separated or Divorced
c. Widowed
d. Living with Partner
e. Single

8. Do you have biological children?
a. Yes (if yes, how many? ___________)
b. No

9. What is the highest grade or level of formal education you have completed?
a. Less than high school
b. High school
c. Some college (or certificate)
d. College graduate
e. Post graduate degree (MA, MS, PhD, MD, DO, etc.)

10. Do you currently practice a religion?
a. No
b. Yes
i. If yes, are you:
Protestant
Roman Catholic
Mormon
Orthodox Greek
Orthodox Russian
Jewish
Muslim
Buddhist
Hindu
Other

Thank you so much for your time. Before we conclude our discussion, is there anything else that you think is important for me to know?

Great, thank you again for your participation. In terms of next steps, you will receive a $50 Visa gift card as an appreciation for being in this research study. The gift card will be sent to you in the mail with a USPS Receipt of Delivery form in which you will acknowledge the receipt of the gift card by signing and returning the form. We do need to collect a little more information from you to process payment.

What is your phone number?
What is your email address?
What is your physical mailing address?

Once the study findings are analyzed, do we have your permission to contact you and send you a community report of the de-identified findings and conclusions from the study?

Wonderful, thank you so much for your time, we really appreciate it.

Material S2 Community Report of Study Findings

Breaking Barriers to Genetic Testing: What We Learned Together

Community Report of Research Findings

The **Breast Health Assessment (BHA)** is an electronic family history tool administered before routine mammograms to identify women at higher risk for inherited breast cancer and increase access to guideline-recommended genetic testing (GT).

***Why* We Did This Study:** Preliminary data revealed that women of African descent who screen high risk and consent to GT via the BHA have a low GT completion rate.

***What* We Did:** Partnered with community members using a community-based participatory research approach to explore barriers & facilitators to GT among at-risk women of African descent

- Designed a qualitative interview study
- Interviewed 12 participants
- Reviewed and interpreted interview findings

***What* We Heard:** Most participants wanted to complete GT and understood its importance for their health and their families. But real-world barriers got in the way.

Process Issues: 50% of participants never received a saliva collection kit for GT

Poor Follow-Up: Some who received kits were not confident to complete next steps

Lack of "Warm Hand-off": Participants wanted an interpersonal touchpoint for support

Cultural Barriers Not An Issue: Mistrust and limited knowledge had no impact on decision

***What* Can Be Done:** Through community input, we identified actionable strategies to address existing gaps in the BHA workflow and improve equitable access to GT

Clear Communication: Reach out before and after appointments to set expectations, and use culturally relevant messaging when providing supportive information

Interpersonal Support: Create more opportunities for human connection

Community Involvement: Continue to work together—because your voices are the key to designing and implementing programs that work

Thank YOU! We deeply appreciate your time, honesty, and trust in sharing your experiences with us.

Sarah H. Choi, MGCS, CGC
(Principal Investigator)
Endeavor Health

Sanjana Ramesh, MPH, PhD
(Co-investigator)
Endeavor Health

Gina Menyah
(Co-investigator)
In The Know, Inc.

Shane Reed, MS
(Research Coordinator)
Endeavor Health

Endeavor Health
